# A simple, robust, broadly applicable insertion mutagenesis method to create random fluorescent protein: target protein fusions

**DOI:** 10.1093/g3journal/jkae036

**Published:** 2024-02-16

**Authors:** Andrew Pike, Cassandra Pietryski, Padraig Deighan, Jason Kuehner, Derek Lau, Anupama Seshan, Paul E March

**Affiliations:** Department of Biology, Oberlin College and Conservatory, 173 W. Lorain St, Oberlin, OH 44074, USA; Department of Biology, Emmanuel College, 400 The Fenway, Boston, MA 02115, USA; Department of Biology, Emmanuel College, 400 The Fenway, Boston, MA 02115, USA; Department of Biology, Emmanuel College, 400 The Fenway, Boston, MA 02115, USA; Department of Biology, Emmanuel College, 400 The Fenway, Boston, MA 02115, USA; Department of Biology, Emmanuel College, 400 The Fenway, Boston, MA 02115, USA; Department of Biology, Emmanuel College, 400 The Fenway, Boston, MA 02115, USA

**Keywords:** insertion mutagenesis, protein fusions

## Abstract

A simple, broadly applicable method was developed using an in vitro transposition reaction followed by transformation into *Escherichia coli* and screening plates for fluorescent colonies. The transposition reaction catalyzes the random insertion of a fluorescent protein open reading frame into a target gene on a plasmid. The transposition reaction is employed directly in an *E. coli* transformation with no further procedures. Plating at high colony density yields fluorescent colonies. Plasmids purified from fluorescent colonies contain random, in-frame fusion proteins into the target gene. The plate screen also results in expressed, stable proteins. A large library of chimeric proteins was produced, which was useful for downstream research. The effect of using different fluorescent proteins was investigated as well as the dependence of the linker sequence between the target and fluorescent protein open reading frames. The utility and simplicity of the method were demonstrated by the fact that it has been employed in an undergraduate biology laboratory class without failure over dozens of class sections. This suggests that the method will be useful in high-impact research at small liberal arts colleges with limited resources. However, in-frame fusion proteins were obtained from 8 different targets suggesting that the method is broadly applicable in any research setting.

## Graphical Abstract

**Graphical Abstract:**
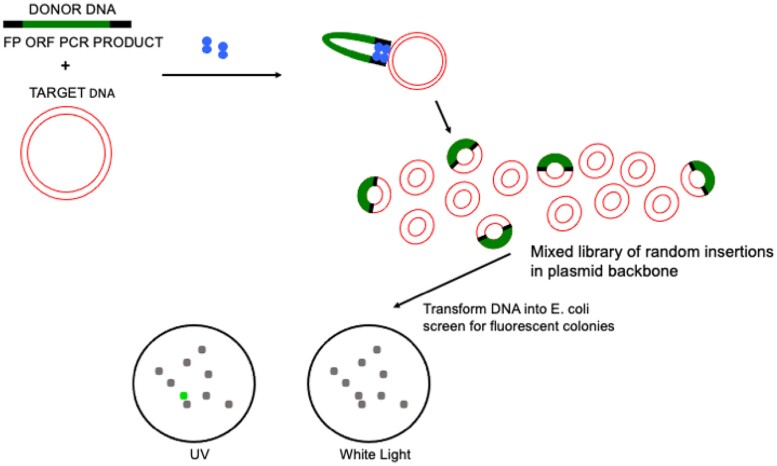

## Introduction

High-impact contemporary research in molecular biology and molecular genetics has traditionally been carried out at institutions with significant budgets for research in labs staffed with graduate students and postdoctoral researchers. Opportunities for undergraduate participation were mostly limited to individual opportunities to apprentice in research labs. Starting in the early 2000s, widespread efforts to remove barriers to research by undergraduates have been initiated. Research was shown to increase the depth of learning and a feeling of inclusion by students (Auchincloss *et al*. 2014). There are still barriers to research by undergraduates. High-impact research in some areas (including molecular biology and molecular genetics) often requires expensive reagents and equipment that may be beyond the reach of some institutions, especially small liberal arts colleges. A method that is simple, inexpensive, and has broad application would help to remove barriers to research. Importantly, a wide-ranging approach should be useful in high-impact research carried out at any level of research at any institution.

Here, we describe an insertional mutagenesis method, dubbed MORFIN (mutagenesis by open reading frame insertion), that gives rise to fully functional mutants tagged with a fluorescent marker. The approach creates open reading frame (ORF) fusions between a fluorescent protein (FP) and a target protein. The method requires 1 in vitro step, and it results in a library of in-frame fusion proteins. Following transformation of this library into *Escherichia coli*, it is possible to screen directly for fluorescent colonies with functional ORF fusions.

## Materials and methods

The method described here depends on the activity of EZ-Tn5 Transposase (EZ-Tn5 is commonly sold in kits for specific purposes; the enzyme by itself, not part of a kit, can be purchased from LGC Biosearch Technologies, Petaluma, CA). This enzyme only requires a 19-nucleotide inverted repeat at each end of a linear blunt-end DNA fragment to catalyze the random insertion of the DNA fragment into a target DNA. PCR primers were designed to create amplicons that contained the 19-nucleotide inverted repeats at the 5′ and 3′ ends of the amplicons (Table 1). A FP ORF was included between the inverted repeats. Primer design included alteration of the FP's ORF start codon and stop codon such that a single continuous ORF was present from the first to the last nucleotide on the amplicon. The details of these DNA primer modifications are shown in Table 1. Any DNA polymerase that results in blunt-end amplicons can be employed in PCR reactions, and in this work, Phusion polymerase (Thermo Fisher Scientific) was employed. In order to increase robustness of PCR reactions using primers with longer “tails,” an initial PCR reaction using TAQ DNA polymerase was employed. Then, 1 µL of the initial PCR reaction was employed in a second PCR using the Phusion polymerase to create high-quality blunt-end amplicons. Amplicons from PCR reactions were purified using QIAquick PCR purification kit.

**Table 1.** PCR primers employed to produce FP ORF amplicons with additional 5′ and 3′ flanking sequences.

Primers used to create amplicons from the mGFPmut3 allele
A. Forward no extra linker: 5′-ctgtctcttatacacatctTGAGTAAAGGAGAAGAAC
B. Reverse no extra linker: 5′ - ctgtctcttatacacatct**A**ATTTGTATAGTTCATC
C. Forward 3 extra linkers: 5′ - ctgtctcttatacacatct**TGGAGCAATCC**AGTAAAGGAGAAGAAC
D. Forward 6 extra linkers: 5′ - ctgtctcttatacacatct**TGCACAAACAGGAGCAATCC**AGTAAAGGAGAAGAAC
E. Reverse plus 3 codons: 5′ - ctgtctcttatacacatct**TCGGACTG**TTTGTATAGTTCATC
Primers used to create amplicons from the mCherry2 allele
A. Forward no extra linker: 5′ - ctgtctcttatacacatctTGGTGAGCAAGGGCG
B. Reverse no extra linker: 5′ - ctgtctcttatacacatct**A**ACTTGTACAGCTCGTCC
C. Forward 3 extra linkers: 5′ - ctgtctcttatacacatctTG**GAGCAATCC**GTGAGCAAGGGCG
D. Forward 6 extra linkers: 5′ - ctgtctcttatacacatctTG**CACAAACAGGAGCAATCC**GTGAGCAAGGGCG
E. Reverse plus 3 codons: 5′ - ctgtctcttatacacatct**TCGGACTG**CTTGTACAGCTCGTCC
Primers used to create amplicons from the mYPET allele
A. Forward no extra linker: 5′ - ctgtctcttatacacatctTGTCTAAAGGTGAAGAATTATTCACTGG
B. Reverse no extra linker: 5′ - ctgtctcttatacacatct**A**ATTTGTACAATTCATTCATACCCTCGG
C. Forward 3 extra linkers: 5′ - ctgtctcttatacacatctTG**GAGCAATCC**TCTAAAGGTGAAGAATTATTCACTGG
D. Forward 6 extra linkers: 5′ - ctgtctcttatacacatctTG**CACAAACAGGAGCAATCC**TCTAAAGGTGAAGAATTATTCACTGG
E. Reverse plus 3 codons: 5′ - ctgtctcttatacacatct**TCGGACTG**TTTGTACAATTCATTCATACCCTCGG

Lowercase text denotes the 19-nucleotide Tn5 recognition element. Uppercase letters denote DNA sequence derived from the FP ORF. Uppercase bold letters denote nucleotides that were altered to adjust the reading frame and inserted to add linker codons. The meaning of “no extra linker,” “3 extra linkers,” and “6 extra linkers” is described in Fig. 4. To create FP amplicons with no extra linkers, primers A and B were employed. To create an amplicon with 3 extra linkers, primers C and B were employed. To create an amplicon with 6 extra linkers, primers D and E were employed.

**Fig. 1.**
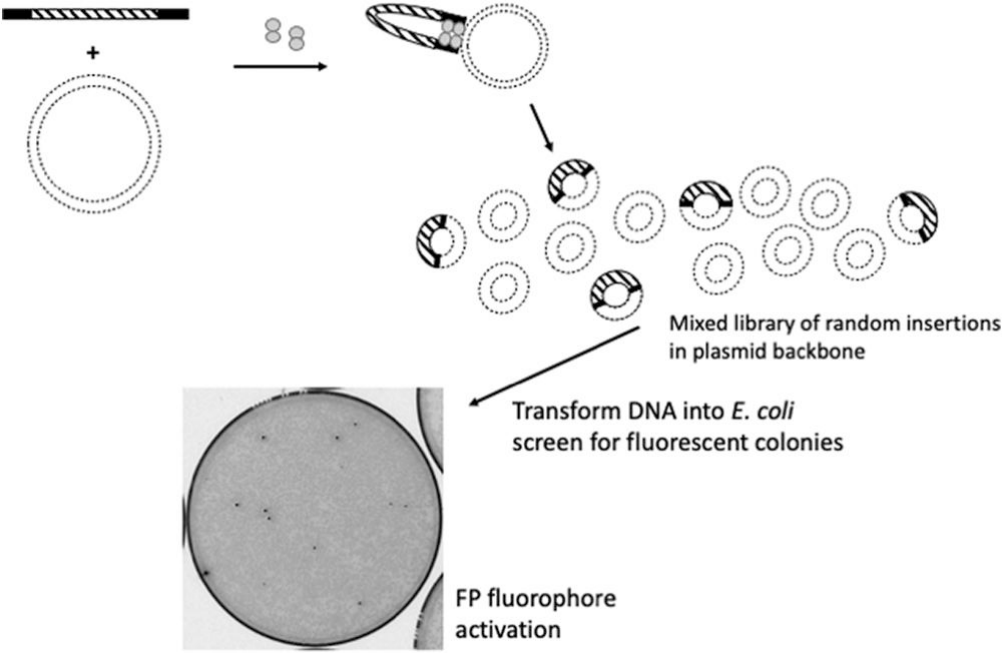
Tn5 Transposase (dumbbell shape) binds to FP ORF amplicons (black and hashed rectangles) at 19-nucleotide Tn5 binding sites (black). This complex interacts with target DNA (dashed circles) at random sites. Following transformation and incubation at 30°C, fluorophore activation allows identification of fluorescent colonies (black spots and gray spots are nonfluorescent colonies).

Target ORFs were contained on *E. coli* expression vectors (Table 2). Purified plasmid DNA was prepared using QIAprep Spin Miniprep Kits. According to EZ-Tn5 manufacturer's specifications, it is essential to use high-quality DNA (both amplicon and plasmid) for the transposition to work. The purity and concentration of DNA samples must be accurately determined. It is important to rigorously follow the manufacturer's instructions for the transposition reaction. Two hundred nanograms of target plasmid must be used, and a molar ratio of amplicon:target DNA of 1:1 is required to ensure that multiple amplicons are not inserted into the same plasmid. The transposition reaction volume was always 10 µL. The reactions were incubated for 2 h at 37°C. Reactions were terminated using 1 µL of Stop solution supplied by the manufacturer and incubated at 70°C for 10 min. Reactions were cooled on ice, and 1 µL of this reaction mixture was used directly to transform *E. coli* MAX Efficiency DH5alpha Competent Cells (Thermo Fisher). Figure 1 shows an overview of the method, and Supplementary File 1 provides a step-by-step protocol.

**Table 2.** Summary of target protein ORFs.

Target ORF	Reason for choosing target ORF	Insertion results	Expression vector
EF G 703 codons	Large, multidomain protein essential for protein synthesis	In-frame insertions always obtained	*lac*-promoter, pBR322 backbone, Amp^R^ (Hou *et al*. 1994)
EF 4 599 codons	A paralog of EF G with 1one less domain	In-frame insertions with efficacy similar to EF G were obtained	*lac*-promoter, pBR322 backbone, Amp^R^ (March and Inouye 1985)
EngA 490 codons	GTP-binding protein with 2 tandem GTP-binding domains	In-frame insertions always obtained but at a frequency lower than for EF G	*lac*-promoter, pBR322 backbone, Amp^R^ (Lee *et al*. 2011)
Era 301 codons	Paralog of EngA except it only contains 1 GTP-binding domain	In-frame insertions obtained but at a 10-fold lower frequency compared to other GTP-binding proteins tested	*lac*-promoter, pBR322 backbone, Amp^R^ (March *et al.* 1988)
FtsZ 383 codons	Known to be recalcitrant to fully functional fusions. Internal in-frame fusions have been constructed using site-directed mutagenesis	A highly restrictive target, correct in-frame insertions only obtained using 6 extra aa linkers with mYPET	*lac*-promoter, pACYC184 backbone, CAM^R^ (Buss *et al*. 2017)
MreB 347 codons	Known to be recalcitrant to fully functional fusions. Internal in-frame fusions have been constructed using site-directed mutagenesis	A highly restrictive target, correct in-frame insertions only obtained using 6 extra aa linkers with mYPET	*lac*-promoter, pACYC184 backbone, CAM^R*^a^*^
Beta-lactamase 377 codons	Antibiotic resistance protein used on most plasmids in this work	No ORF fusions obtained	All pBR322 plasmids
CAT 219 codons	Antibiotic resistance protein used on some plasmids in this work	ORF fusions obtained that are fully functional	See FtsZ and MreB
LacI 360 codons	Included in *lac*-promoter expression vectors	In-frame fusions are very rarely obtained	All vectors employed in this work contained this ORF

Transposition reactions were employed in transformations into ultracompetent *E. coli* directly without any further treatment. Fluorescent colonies were purified by restreaking, and plasmids were obtained from fluorescent colony clones. These plasmids were subjected to DNA sequence analysis to confirm the nature of the fused ORF obtained.

^*a*^MreB expression vector was provided by the Thomas Bernhardt lab, Harvard Medical School.

Transformations were incubated according to the manufacturer's instructions, and 100 µL was plated onto each of 10 plates. The final volume of the transposition reaction was 11 µL, enough for 11 transformations resulting in 110 plates per transposition if desired. This procedure is designed to maximize the number of colonies screened. Transformations must be plated at high colony density (5 × 10^2^ per plate). Plates containing 1 × 10^2^ colonies per plate or less do not provide enough candidates to recover fluorescent colonies. This is why we employ MAX Efficiency DH5alpha competent cells, but any strain with comparable transformation efficiency can be used (>1 × 10^9^ transformants/µg plasmid DNA). It is essential to incubate the plates at 30°C or less (room temperature works well). We have never obtained fluorescent colonies from 33 or 37°C incubations. Furthermore, positive candidates identified at 30°C that are regrown at 37°C will not be fluorescent. Fluorescent colonies were restruck and incubated at 30°C to purify positive clones. Plasmid DNA from these cells was purified and employed to determine the site of the insertion of the FP ORF by DNA sequencing.

DNA sequencing was performed using a “universal” sequencing primer strategy. The primer was designed to hybridize about 60 nucleotides from the 3′ end of the FP ORF and direct sequencing toward the 3′ end of the ORF, across the junction site and into the flanking target gene. Such a primer would allow confirmation of the presence of the FP, the maintenance of the correct reading frame at the junction, and the identification of where the insert is located within the target. We did not assess the 5′ junction because it must be correct for the expression of a fluorescent product. DNA sequencing was performed by Quintara Biosciences, Cambridge, MA.

## Results and discussion

The FP PCR amplicon was designed to contain no start codon, no stop codon, and a single ORF from end to end. Therefore, in order for a FP to be expressed, the FP ORF must be inserted in the correct orientation between adjacent codons to preserve the reading frame. The subsequent colony screen eliminates all insertions that land in the plasmid backbone, are in the incorrect orientation, or are out-frame insertions. The expected frequency of positive fluorescent colonies can be estimated. The manufacturer of EZ-Tn5 suggests that about 1 plasmid in 200 will contain a single insertion. The ratio of the target gene size to the total plasmid size influences the frequency (in our experiments with the EF G ORF, this ratio is 2,109 bp/6,837 bp). The orientation of the inserted FP ORF relative to the transcription and translation of the target ORF was random so the frequency of a fluorescent positive colony was reduced by an additional one-half. The probability of insertion between codons (not within a codon) must be accounted for (1 out of 3). Other factors that cannot easily be controlled for but can affect the frequency of positive colonies include the expression level of the fused ORF, and target protein structure (for example proteins containing multiple independent domains would be expected to offer more sites for successful insertion). By multiplying the factors for which there were numbers, the expected positive colony frequency for the case of the EF G expression plasmid employed here was 1 positive colony for every 3890 screened. The observed frequency of in-frame fusions (Supplementary Table 1) was much higher, suggesting that for this ORF, uncontrolled factors (expression level of the fused ORF and target protein structure) significantly affected the frequency of obtaining in-frame fusions and fluorescent colonies. In the case of the Era ORF (43% of the EF G ORF), in-frame insertions occurred at more than 10-fold lower compared to EF G (Supplementary Table 2).

**Fig. 2.**
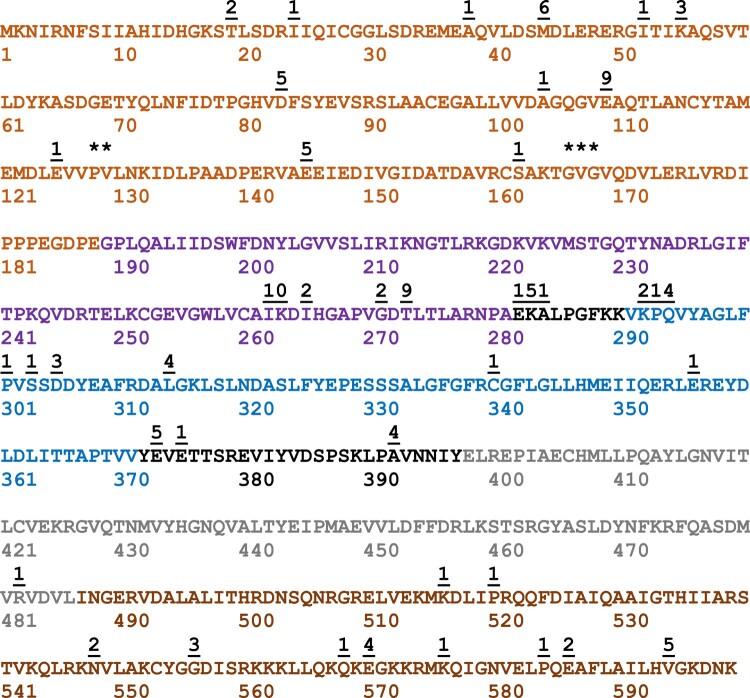
The cumulative data obtained from undergraduate laboratory class are shown. From 2014 to 2020, a single target ORF (EF 4) was employed in transposition reactions with the FP ORF GFPmut3. The amino acid sequence of the EF 4 ORF is shown. The colored amino acid residues indicate EF 4 domains: domain 1 (tan residues 1–188), domain 2 (purple residues 189–281), domain 3 (blue residues 291–371), domain 4 (gray residues 398–486), and the C-terminal disordered domain (brown residues 487–599). The underlined numbers above the sequence indicate insertion sites that were confirmed by student researchers and the number of times each insertion site was recovered. ** indicates the insertion at codon 128 was recovered 20 times and codon 129 twice. *** indicates a cluster of insertions at codons 167 (twice), 168 (13 times), and 169 (once). The cluster at 282, 283, and 284 was recovered once, 5 times, and once, respectively, and at 292, 293, and 294, twice, once, and 4 times, respectively. These data were derived from 159 separate student experiments.

To demonstrate the simplicity and robustness of this method, we employed it in an undergraduate laboratory class that included all biology majors with a very wide range of laboratory training. Between 2014 and 2020, 511 students in both spring and fall semesters undertook the experimental protocol detailed in the class lab manual (see Supplementary File 1). To monitor robustness across multiple class sections in different semesters, the undergraduate laboratory class module was conducted using only 1 target ORF (EF-4) and 1 transposable amplicon containing the GFPmut3 ORF. All class sections across all years successfully obtained fused ORFs for each student for further study. Figure 2 provides a map of all insertion sites documented between 2014 and 2020. Some domains (1 and 2) were particularly good targets for insertion whereas others were very rarely targeted (only a single insertion was ever observed in domain 4). This distribution of insertion sites would not be possible to predict by an ab initio approach highlighting the usefulness of the method: the ability to generate a very large library of useful fusion proteins.

In parallel, to demonstrate the broad applicability and potential of the method, investigations were undertaken to explore the effect of different FP ORFs and the effect of the linker sequence between the target and the FP ORF. Finally, the approach was also used on several different target ORFs. Three specific questions have been addressed: (1) does alteration of the sequence that flanks the FP ORF affect the efficacy of successful transposition; (2) can other FPs replace the GFPmut3 allele; and (3) is it possible to apply MORFIN to other target genes?

**Fig. 3.**
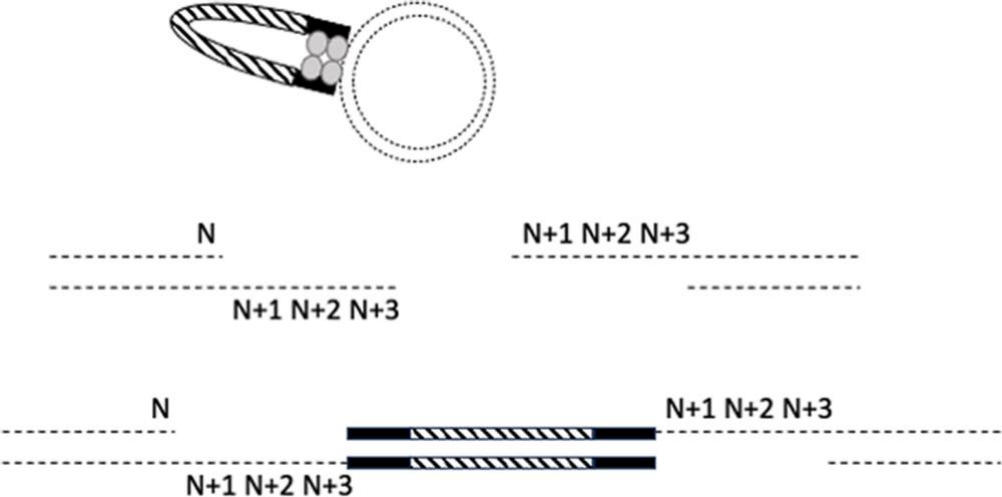
Tn5 Transposase (gray dumbbell color) binds randomly to the target DNA (dashed circle) and cuts target DNA strands at sites 9 nucleotides apart generating a 9-nucleotide complimentary overhang. The transposase ligates the inserted DNA generating a double-strand structure with 5′ and 3′ 9-nucleotide gaps. The gaps in the resultant double-strand circle are filled in by host DNA repair polymerases following transformation. Codon N is the codon that immediately precedes the Tn5 cut site. Codon N + 1, N + 2, and N + 3 represent the 3 codons following N, which comprise the 9-nucleotide overhang. The result of ligation by Tn5 and host cell repair is that codons N + 1, N + 2, and N + 3 were repeated on the 3′ side of the FP ORF (hashed rectangle). The 19-nucleotide recognition sequence for Tn5 is represented by the black rectangle. Only cleavages between codons are recovered because cleavage within a codon produces an out-of-frame ORF. Out-of-frame ORF fusions fail the colony screen for fluorescence.

**Fig. 4.**
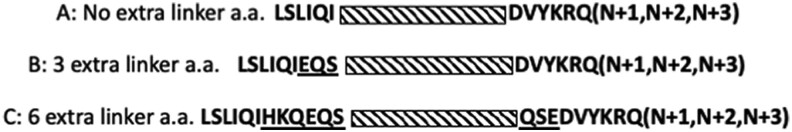
Testing effects of altering linker sequence (black bold capital letters) between the target ORF and the FP ORF (hashed rectangle). a) The 19-nucleotide Tn5 recognition element is a defined sequence that must always be present at the 5′ and 3′ ends of the inserted DNA as inverted repeats. The first 18 nucleotides would encode the amino acid sequence LSLIQI, and the 19th nucleotide would become the first base of the first codon of the FP ORF. At the 3′ end, the first nucleotide of the inverted repeat would replace the third nucleotide of the ORF's stop codon creating a read-through ORF. The subsequent 18 nucleotides encode the amino acids DVYKRQ. Due to the staggered cut of the Tn5 transposase, codons N + 1, N + 2, and N + 3 were repeated at the 3′ end of an insertion (Fig. 3) comprising additional nonnative sequence. b) To make the number of 5′ and 3′ inserted codons symmetrical, 3 additional codons were inserted at the 5′ end (underlined letters represent the amino acids encoded by this modification). c) To increase the length of the linker region to optimal 12 codons and insert codons encoding more favorable linker amino acids, codons were added, which would encode the underlined amino acids. George and Heringa (2002) and Suyama and Ohara (2003) were used as a guide to design optimal linker length and favorable interdomain amino acid linker sequence. PCR primers employed to insert these modifications are listed in Table 1.

The sequence that flanks the FP ORF must include the DNA sequence required for Tn5 transposition, but additional codons can be included in linker sequences. EZ-Tn5 requires a specific 19-nucleotide inverted repeat at the 5′ and 3′ ends of a DNA sequence destined for transposition (Goryshin and Reznikoff 1998). The 19-nucleotide sequence represents a binding site for the Tn5 transposase (Fig. 1). Insertion into target DNA results from a double-strand cleavage, which is staggered with a 9-nucleotide overhang. Due to this, 3 codons at the 3′ end of a cleavage site are a repeat of the same codons found at the 5′ end (Fig. 3). Therefore, the 5′ flanking sequence would contain 6 spacer codons between the target protein ORF and the FP ORF, whereas the 3′ flanking sequence would contain 9 extra codons (Fig. 3). Although the 19-nucleotide recognition sequence cannot be altered, it is possible to insert additional codons between this sequence and the FP ORF. Investigations into the optimal lengths of linker sequences between protein domains and the composition of those sequences guided our initial experiments (George and Heringa 2002; Suyama and Ohara 2003). Based on these data, we created a linker to insert 3 extra codons at the 5′ end to balance the number of codons at each end to 9 extra codons. In addition, linkers were created to include 12 extra codons at each end (Fig. 4 and Table 1).

**Fig. 5.**
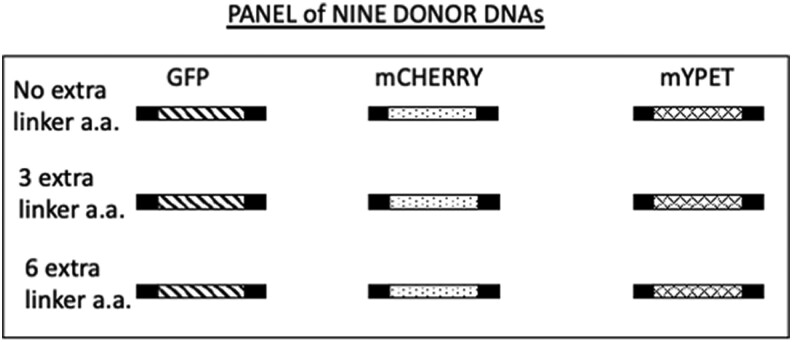
Schematic of donor amplicons generated as substrates for MORFIN mutagenesis. The black rectangle indicates the position of 5′ and 3′ linker sequence. “No extra linker a.a.,” “3 extra linker a.a.,” and “6 extra linker a.a.” are explained in Fig. 4. The diagonal filled rectangle represents the ORF for GFPmut3, the dotted rectangle represents the ORF for mCherry2, and the hashed rectangle represents the ORF for mYPET.

This linker set was employed to create amplicons derived from the ORFs of 3 different FPs, GFPmut3, mCherry2, and mYPET (Fig. 5). This panel of amplicons was used in initial experiments to examine effects associated with altering either the linker sequence or the FP ORF.

**Fig. 6.**
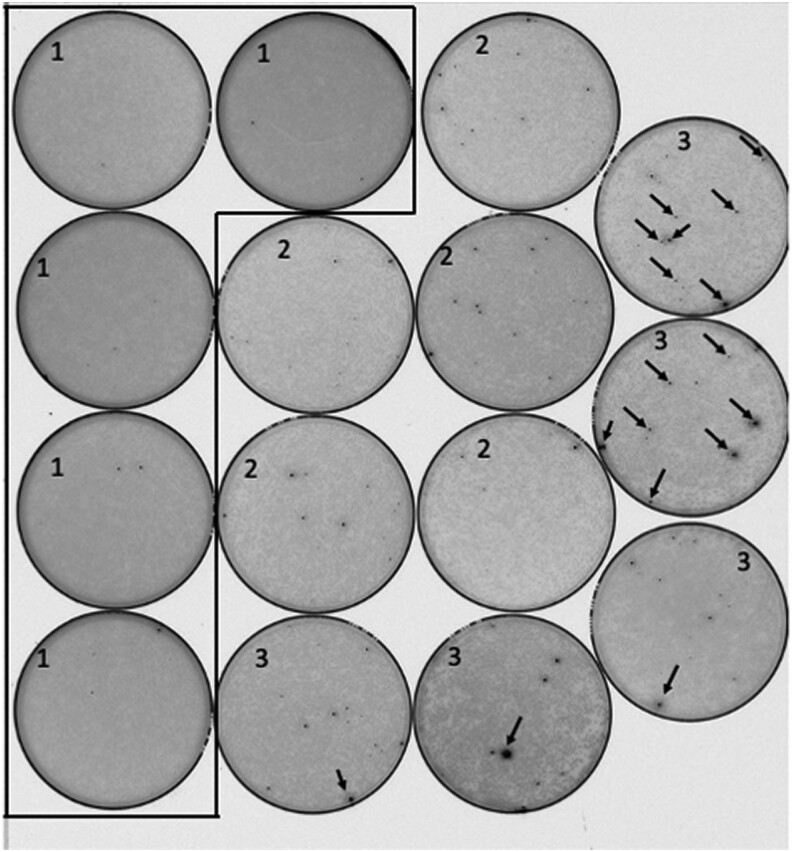
The unexpected sensitivity of mYPET to linker sequence. Transposition reactions were transformed directly into *E. coli* and plated at high colony density. After a 36-h incubation at 30°C, the plates were imaged using a laser plate scanner. Nonfluorescent colonies are light gray, and fluorescent colonies are black. Examples of prominent black fluorescent colonies are indicated by the arrowheads. The results from 3 different amplicons are shown: (1) mYPET with no extra linker, (2) mYPET with 3 extra linkers, and (3) mYPET with 6 extra linkers. The meaning of “no extra linker,” “3 extra linkers,” and “6 extra linkers” is explained in Fig. 3. The target ORF in this case expressed EF G. All black colonies were restruck to purify clones that produced FPs. Purified clones were subjected to plasmid purification, and pure plasmids were sequenced to confirm insertion sites. The tiny black specks found on plates numbered 1 were not fluorescent bacterial colonies.

**Fig. 7.**
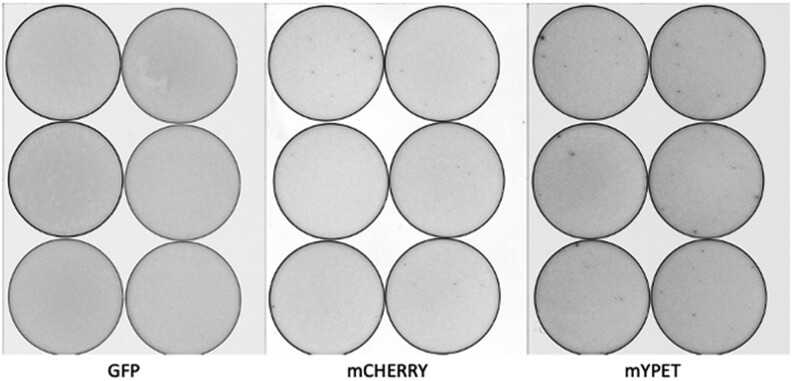
An example of the restrictive nature of the FtsZ ORF. Transposition reactions were transformed directly into *E. coli* and plated at high colony density. After a 36-h incubation at 30°C, the plates were imaged using a laser plate scanner. Nonfluorescent colonies are light gray, and fluorescent colonies are black. The results from 3 different FPs are shown. In each case, the linker was the 6 extra linker (Fig. 3). Fluorescent colonies were only found when mCherry or mYPET was employed as the FP ORF. All of the mCherry candidates were off target. In-frame FP–FtsZ fusions were only obtained from mYPET transposition reactions. The same result was obtained for the MreB ORF. All black colonies were restruck to purify clones that produced FPs. Purified clones were subjected to plasmid purification, and pure plasmids were sequenced to confirm insertion sites.

Table 2 lists the ORFs that were tested using the amplicons created. It was beyond the scope of this study to test the entire panel of amplicons in all the ORFs listed in Table 2. The reason for this is that 9 transpositions are required to test the entire collection of amplicons. Each plate screen requires a minimum of 10 plates to obtain a collection of fluorescent-positive colonies (often more than 10). Accounting for different frequencies of positive colonies, testing the entire panel would require about 10^3^ plates. The EF G ORF, the FtsZ ORF, and the MreB ORF were tested using the entire panel of amplicons. The EF G ORF yielded fluorescent colonies and in-frame fusions from all combinations of linkers and FPs except 1 combination: mYPET and no extra linker amino acids (Fig. 6). In fact, we never obtained any positive candidates from that combination, suggesting that the mYPET FP is particularly sensitive to flanking sequence. Additional evidence of the linker spacer effect on mYPET fusions is apparent in Fig. 6. When mYPET was combined with 6 extra aa linkers, fluorescent colonies were observed of varying signal intensity. A more intense signal would be a consequence of lower fusion protein turnover, a higher percentage of correctly folded expressed protein, or both. The mYPET 6 extra aa linkers also allowed for fusion proteins to be obtained for ORFs that were otherwise recalcitrant to the MORFIN approach. The FtsZ and MreB ORFs only gave rise to positive candidates with in-frame fusions with 1 combination: mYPET with 6 extra linker amino acids (Fig. 7). Using homology modeling of MreB, Bendezu *et al*. (2009) identified a surface loop centered around codon 228 and employed site-directed mutagenesis to create a functional internal fusion to mCherry. Among the MreB candidates from this work, we identified mYPET fusions after codon 228 and nearby at 235. Site-directed mutagenesis was employed to investigate internal FP fusions to FtsZ (Moore *et al*. 2017). From their collection, 1 fully functional insertion was obtained between FtsZ codons 55 and 56. In this study, an insertion was obtained after codon 57.

It is important to consider protein production levels because if an in-frame fusion is expressed at a low level or is rapidly turned over, it would fail to be detected in the colony screen. FtsZ presents a particular challenge because expression levels are tightly regulated in dividing cells. For this reason, a plasmid backbone with a lower copy number was used (Table 2). The antibiotic marker gene on plasmids expressing the FtsZ and MreB ORFs was CAM^R^ conferring resistance to chloramphenicol. Although the chloramphenicol acetyltransferase (CAT) ORF was not designed as a target in these studies, in-frame fusions to the CAT ORF were obtained in the fluorescent colony screen (1 after codons 20, 208, and 214, and 9 after codon 6). These were off target but informative in-frame fusions. They were confirmed by plasmid purification, retransformation, and DNA sequencing. Structural analysis of insertions sites showed that the insertions were located at the external face of monomers and could be accommodated within a homotrimer structure. These fusions were both fluorescent and conferred resistance to chloramphenicol, confirming that both partners of the fused protein were functional. The CAT ORF is only 219 codons, so it represents a small target. In addition, the functional CAT protein must assemble into a homotrimer (Leslie *et al*. 1988). It was not expected that a FP ORF (with the number of codons similar to the target ORF) could be inserted into the CAT ORF and that a functional trimer could assemble. This observation strongly supports the broad application for this technology and its potential to recover fully functional protein chimeras; however, the gene encoding CAT should not be employed as a selectable plasmid marker for the MORFIN approach because off-target insertions were not rare. In-frame fusions were not ever seen within the beta-lactamase ORF (this observation includes hundreds of fusions examined in the undergraduate laboratory classes). Beta-lactamase is a secreted protein, and it is not a surprise that proteins that cross a membrane would not easily accommodate a FP fusion with a functioning fluorophore. ORF expression plasmids using Amp^R^ as the selectable marker are preferable in the MORFIN approach.

A consideration regarding the target ORF is that the frequency of obtaining positive FP–target protein fusions depends on the size of the target ORF and the domain structure of the target protein. In an effort to investigate the effect of domain structure on insertion frequency, a panel of related GTP-binding proteins was tested (Table 2; EF G, EF 4, EngA, and Era). Each of these 4 ORFs was present on the same plasmid backbone and targeted by the FP amplicon containing the FP mGFPmut3 with no added linker aa. EF G, like EF 4, is a large multidomain protein and gave rise to a positive ORF insertion frequency of 70 inserts/40,000 transformants screened (Supplementary Table 1). The EF G ORF is 703 codons, and the protein contains 6 distinctly folded domains (Czworkowski *et al*. 1994). The EF 4 ORF is 599 codons, and the protein contains 5 domains (Evans *et al*. 2008). EngA and Era (Table 2) are related GTPases whose ORFs contain 3 and 2 domains, respectively (Robinson *et al.* 2002; Chen *et al*. 1999). The Era ORF contains 301 codons and was found in fluorescent fusions 10 times less frequently than EF G. The EngA ORF contains 490 codons, and it was found at frequency about 5 times higher than Era (Supplementary Tables 1–3). One recurring observation across all 4 ORFs was that the GTP-binding domain was frequently a target for in-frame FP insertions, so this method would be useful to explore biochemical and cellular functions of GTP-binding proteins.

One important consideration in selecting a fluorophore protein is that the mYPET ORF gives rise to a FP that must be detected by laser activation. This means that access to expensive detection devices (such as a laser plate scanner) is necessary. The mGFPmut3 ORF product can be detected with inexpensive handheld UV devices. Colonies that express mCherry2 can be identified because they turn red even in ambient room light. These considerations may be critical for implementation into undergraduate curricula and research.

In conclusion, the MORFIN method is a simple and powerful research tool. Transformation of transposition reactions with no intervening steps eliminates time and resource-intensive steps traditionally employed in methods to create FP fusions (Biondi *et al*. 1998; Sheridan *et al*. 2002; Gregory *et al*. 2010; Moore *et al*. 2017). The power of the method arises from subsequent fluorescent colony screen. The transposed DNA must land within an ORF, in the correct orientation, reading frame, and be expressed highly enough for a fluorescent signal to arise. The fluorophore of FPs is exquisitely sensitive to the structure of the protein (Remington 2011). Since the colonies are fluorescent, the fluorophore must be precisely and correctly folded. It is extremely likely that the surrounding target protein is not misfolded because prior research demonstrates that GFP fluorescence is negatively impacted when it is flanked by misfolded sequence (Waldo *et al*. 1999; Ito *et al*. 2004; Kim and Hecht 2005; Pedelacq *et al*. 2002; van den Berg *et al*. 2006; Wurth *et al*. 2002; Yang *et al*. 2003). Correct folding is supported by the evidence presented here that fully functional fusions in the CAT ORF were obtained. Furthermore, the plate screen apparently strongly screens out fused ORFs that would disrupt the protein structure. In all cases where detailed structural mapping has been done (EF G, EF 4, Era, and CAT ORFs), all of the insertions are localized at external surfaces of the protein structure. The activities described here are perfectly suited to provide students with experience in a variety of commonly used molecular biology methods. The module presented in the laboratory manual (see Supplementary File 1) is designed to give students ownership in the project by providing each student with a random, unique insertion site to characterize. Students must utilize critical thinking skills to develop appropriate predictions of the results and to interpret the data from the experiments. MORFIN can additionally be used to quickly create a large, mixed library of random mutations to screen for promising candidates that can be investigated in a continuing research program. Although we only tested ORFs derived from *E. coli* genes, we expect MORFIN to be applicable to ORFs from any source if the ORF's product can be expressed in the *E. coli* cytoplasm.

## Supplementary Material

jkae036_Supplementary_Data

## Data Availability

Strains and plasmids are available upon request. In addition to insertion sites identified within the text for EF 4 and CAT, Supplementary Table 1 identifies all insertion sites for EF G. Supplementary Table 2 identifies all insertion sites for Era and EngA. Supplementary Table 3 identifies all insertion sites for FtsZ and MreB. Supplemental material available at G3 online.
