# Supplementary material for: A simple, robust, broadly applicable insertion mutagenesis method to create random fluorescent protein: target protein fusions
Source: G3 (Bethesda). 2024 Feb 16;14(5):jkae036. doi: 10.1093/g3journal/jkae036 (PMC11075570; doi:10.1093/g3journal/jkae036)
Supplement: jkae036_Supplementary_Data [file jkae036_supplementary_data.zip › File_S1_G3-2024-404874.docx]

**File S1. Supplementary Material**

**Class Lab Manual Step – by – Step Protocols Using the MORFIN Method**

**Table of Contents**

| Step 1 | Purification of target LepA/EF-4 plasmid DNA | 2 |
| --- | --- | --- |
| Step 2 | PCR of GFP donor DNA | 2 |
| Step 3 | Purification and characterization of PCR DNA | 3 |
| Step 4 | Transposition reaction | 4 |
| Step 5 | Transformation of Transposition | 5 |
| Step 6 | Identification of GFP+ colonies | 6 |
| Step 7 | Confirmation GFP positive strains/Inoculation | 7 |
| Step 8 | Candidate Plasmid DNA Isolation | 8 |
| Step 9 | Restriction Digestion/ Sequencing | 8 |

**Experimentation Schedule.**

***Overview:*** Initially the target plasmid (pPEM109) containing the *lepA* gene will be isolated and donor transposon DNA will be synthesized. After characterizing these pieces of DNA, they will be combined with Tn5 transposase. This reaction will generate an insertion library as illustrated in FIGURE 1. This library will be transformed into *E. coli* in order to isolate insertion-positive candidates by screening for fluorescent colonies growing on plates. The plasmid derived from fluorescent colonies will be purified and compared to the original target DNA. The characterization will include determining the relative sizes of the plasmid DNA, and the EF-4 protein produced, DNA sequence analysis, and western blotting to prove that the insertion-positive candidate contains GFP sequence. DNA sequence information will be used, in conjunction with information about the three-dimensional protein structure to determine where the GFP sequence is inserted within the structure of EF-4. Functional assays will be performed to assess whether the newly created GFP fusion proteins are active.

**STEP 1:** **PURIFICATION OF TARGET LEPA/EF-4 PLASMID DNA**.

**Purpose:** To amplilfy and purify plasmid pPEM109, containing the *lepA* gene as a GFP target, for an *in vitro* Tn 5 transposase-catalyzed transposition reaction

**Procedure:**

Plasmid Purification.

**Each student will start with 1.5 mL of cultured *E. coli* containing plasmid pPEM109.**

1. Obtain a microfuge tube containing 1.5 mL of saturated overnight *E. coli* culture (containing desired plasmid pPEM109). Label top of tube with your initials.

2. Purify the plasmid using an appropriate miniprep or similar kit according to the manufacturer’s instructions.

3. The DNA will be stored at -20^°^C until the next lab meeting.

**-------------------------------------------------------------------------------------------------------------------**

**STEP 2: GENERATION OF TRANSPOSITION REACTION “DONOR” DNA USING PCR.**

**Purpose:** To use the Polymerase Chain Reaction (PCR) to create a linear double-stranded piece of DNA that can serve as the “donor” DNA in an *in vitro* Tn5 transposase-catalyzed transposition reaction.

**Procedure:**

1. Each student should label a single PCR tube with their individual initials.

*Note: The PCR tubes are different than microfuge tubes. They are smaller to better fit into the thermocycler machine and have have thinner walls more conducive for heat conduction.*

2. Add the reagents to PCR tubes on ice in the order shown below to prepare the following 50 uL reactions:

*HINT: Put a checkmark* ✓ *next to protocol reagents “Reagent added?***”** *as you add them to help you remember what has been added to each tube.*

| **Reagents** | **PCR reaction**  **(uL)** | **Reagent added? (**✓**)** | **Final**  **Concentration** |
| --- | --- | --- | --- |
| ddH_2_0 | 32.5 |  |  |
| 5X HF Buffer | 10.0 |  | 1X |
| 10 mM dNTPs | 1.0 |  | 200 uM |
| GFP-F primer (10 uM) | 2.5 |  | 0.5 uM |
| GFP-R primer (10 uM) | 2.5 |  | 0.5 uM |
| pGLO Plasmid DNA template | 1.0 |  | <250 ng |
| Phusion DNA Polymerase enzyme | 0.5 |  | 1 unit/50 uL |
| **Total:** | **50** |  |  |

3. Seal the PCR reaction tubes with caps, making sure they are tightly closed. Mix the reaction by tapping the side of the tube. Spin down in a mini microcentrifuge to collect contents.

4. Keep your reactions on ice until ready to load into the PCR machine that has been programmed as shown below:

Cycling conditions for GFP PCR

1) 98°C, 30 sec.

2) 98°C, 10 sec.

3) 62°C, 20 sec

4) 72°C, 30 sec

5) Repeat 2-4, 30X

6) 72°C, 5 min.

7) 10°C, Hold

**-------------------------------------------------------------------------------------------------------------------**

**STEP 3: PURIFICATION & CHARACTERIZATION OF PCR PRODUCT (“DONOR DNA”).**

**Purpose:** To check that the PCR product was produced, purify the PCR product, and determine the concentration of the PCR product

**Procedure:**

PCR Clean-up

1. Clean up the plasmid using PCR cleanup kit according to the manufacturer’s instructions.

Nanodrop

Use 2 ul of your cleaned up PCR sample to determine the DNA concentration using a Nano-Drop spectrophotometer. Your lab instructor will demonstrate how to use the Nano-Drop spectrophotometer. Record this value in your lab notebook, including concentration (ng/uL) and 260/280 purity ratio.

Agarose Gel Electrophoresis

1. Remove 5 uL of your cleaned-up PCR reaction and add into a new microcentrifuge tube along with 3 uL of loading dye. Pipette up and down to mix. **Note: Do not add dye to your entire PCR SAMPLE**!

2. Load the 8 uL sample into a 1% agarose gel containing fluorescent dye and perform electrophoresis. Save the remainder of the cleaned-up PCR for future use.

**DATA ANALYSIS**

1) Were you successful in amplifying the desired PCR product? How can you tell? Be sure to describe any expected bands and relative sizes.

2) Compare the yield of your reaction compared to your partner’s reaction. What could account for any differences that you observe?

3) What sequences are contained within your PCR product, and how will the PCR DNA be used in future experiments?

4) Consider any potential sources of error in the experiment (even if not observed) and how that may have affected the outcome.

**-------------------------------------------------------------------------------------------------------------------**

**STEP 4: TRANSPOSITION REACTION.**

**Purpose:** To set up the transposition reaction with GFP PCR donor DNA and pPEM109 plasmid DNA

**Procedure:**

Transposition Reaction: Tn5 transposase is expensive, but one 10 microliter reaction is enough for 8 pairs of lab partners. The reaction will be set up by the instructor and shared with students.

1. The transposition reaction requires 200 ng of plasmid pPEM109 (target DNA) and a 1:1 molar ratio of donor transposon (source of the GFP gene). The students will have to calculate the volume of plasmid pPEM109 DNA, GFP DNA, and water that are required. Then, set up the transcription reaction as described in the table below

| **Reagents** | **Restriction enzyme**  **reaction**  **(uL)** | **Reagent added? (**✓**)** |
| --- | --- | --- |
| 200 ng plasmid pPEM109 (target) DNA | X |  |
| GFP (donor) DNA | Y |  |
| ddH_2_0 | Z |  |
| 10X Buffer | 1.0 |  |
| EZ Tn5 transposase | 1.0 |  |
| **Total:** | **10** |  |

2. Incubate the reaction for 2 hours at 37°C.

3. Terminate the reaction by adding one microliter of 10X stop solution and incubating for 10 minutes at 70°C.

At this stage the sample can be stored at -20 degrees C. Each lab partner pair will use one microliter of this reaction mixture in STEP 5 to transform ultra-competent *E. coli* cells.

**-------------------------------------------------------------------------------------------------------------------**

**STEP 5:** **TRANSFORMATION OF TRANSPOSITION MIXTURE INTO *E. coli*;**

**Purpose:** To introduce a mixed library of GFP insertion plasmids into bacteria to measure GFP fluorescence *in vivo*

**Procedure:**

**Important note:** Correct handling of High Efficiency Competent cells is crucial to obtain the maximum number of transformants. Cells must be removed from -80 freezer and placed directly in ice. Cells must be thawed gently on ice. Maintain ice temperature until heat shock. For heat shock be exact with the incubation time and be sure cells are transferred from ice directly to 42 degrees. Do not alter incubation times.

1. Thaw *E. coli* strain DH5alpha-F’lacIQ in ice bucket.

2. Label a sterile 1.5 mL tube with your initials and “+library”. Add 1 ul of transposition reaction mixture into the labeled tube and place on ice.

3. Observe the *E. coli* DH5alpha-F’lacIQ cells and give the tube a quick flick to be sure cell suspension is uniform. Tap tube on bench to collect cells and place back on ice. DO NOT ALLOW CELLS TO WARM UP DURING TRANSFER. Try to be precise and quick with your transfer. Add 100 ul of thawed cells to the tube containing 1 ul of transposition library.

**NOTE:** AT THIS POINT IN THE PROCEDURE IT IS CRITICAL THAT SAMPLES BE MAINTAINED ON ICE.

4. Incubate your sample tube (containing 1 ul of transposition mixture and 100 ul of cells) on ice for 30 min.

5. Heat shock cells by transferring sample tube from ice directly into a 42^o^C heating block. Incubate for EXACTLY 45 seconds.

6. Transfer sample tube directly from heating block back to ice bucket. Incubate 2 minutes on ice.

7. Add 0.9 mL of SOC medium (maintained at room temperature) to the cell mixture.

8. Place sample tube in a rotator in a 37°C incubator for 1 hour.

9. Spread cells from sample tube onto LB+Amp plates by aliquoting 0.1 mL onto each plate and spreading with sterile beads. You should generate 10 plates from this step. Plates should be labeled with group initials and the date.

10. Incubate plates in a lab drawer (at room temperature) until next meeting.

***Note:*** *4 days at room temperature will yield colonies but they may be small depending on ambient temperature (20-25°C). Switch plates to 30°C for final overnight incubation the day prior to next lab meeting.*

**-------------------------------------------------------------------------------------------------------------------**

**STEP 6:** **IDENTIFICATION AND RE-STREAKING OF GFP POSITIVE COLONIES**

**Purpose:** To identify GFP positive transformant colonies and obtain genetically pure isolates for additional characterization

**Procedure:**

1. Inspect plates using hand held UV light, carefully inspecting all colonies for signs of fluorescence. Circle the back of the plate with a marker if GFP positive colonies are identified.

2. Record number of ALL green glowing colonies. Record total number of colonies by counting section and estimating for remainder of plate. Estimate frequency of GFP+ colonies over total transformants. If you have no colonies record that. If you have a confluent lawn of cells record that as a “lawn”. Your instructor will assist you with this.

3. Each student should re-streak one GFP positive colony onto a new LB Amp plate. If you obtained more than one green colony, pick the strongest GFP positive colony that is well separated from neighboring colonies. If you have no positive colonies borrow colonies from colleagues that have extra. Carefully pick GFP positive colonies, as instructed. Consult instructor about discarding transformation plates.

4. LABEL EACH ISOLATE WITH A DISTINCT NUMBER AND RECORD YOUR LABEL.

5. Incubate re-streak plates at room temperature until next meeting.

*Note to instructor: Keep several plates that have multiple GFP-positive colonies as a backup in case the re-streaked plates don’t grow properly.*

**DATA ANALYSIS**

1) Was the transformation of the mixed plasmid library successful? Why or why not?

2) What are 2 different transposition events that could result in a GFP positive colony? What are 2 different transposition events that could result in a GFP negative colony?

3) Based on data up to this point, can you tell if the transposition and transformation was successful in generating a successful *lepA* gene-GFP gene ORF fusion strain? Why or why not?

4) Consider any potential sources of error in the experiment (even if not observed) and how that may have affected the outcome.

**-------------------------------------------------------------------------------------------------------------------**

**STEP 7: CONFIRMATION OF GFP POSITIVE STRAINS AND INOCULATION OF POSITIVE CANDIDATES INTO LIQUID MEDIA**

**Purpose**: To confirm we have genetically pure GFP positive isolates and start liquid cultures for future analysis of plasmid DNA sequence and GFP protein expression

**Procedure:**

1. Lab partner pairs should have two plates from STEP 6. Each plate contains a re-streak of an original colony that was GFP-positive. Inspect plates to confirm GFP-positive colonies.

2. Record whether there is growth on each plate and record whether or not there are GFP-positive colonies. Consult with an instructor to determine your official candidate ID number (each student will have their own).

3. Each student pick one GFP positive colony using a sterile loop. If there is growth on both plates from, be sure that one colony is taken from each plate. If there is growth on only one plate or no growth on either plate, then consult with instructor to formulate a plan). Each GFP-positive colony should be transferred into one 15 mL sterile conical tube containing 1.0 mL of LB Amp. Incubate this tube at 30^°^C during the lab.

5. Incubate the conical tubes at 30^°^C overnight and arrange with your instructor to transfer these cultures to 4^°^C the following morning.

SAVE ALL GOOD RESTREAK PLATES IN CASE WE HAVE TO GO BACK TO THEM.

LABELING IS IMPORTANT!

**-------------------------------------------------------------------------------------------------------------------**

**STEP 8: CANDIDATE PLASMID DNA ISOLATION**

**Purpose:** To isolate pPEM109+GFP plasmid and total protein from liquid cultures containing the GFP-positive transformant

**Procedure:**

PLASMID PURIFICATION:

1. Vortex to resuspend the cells from your 3 mL culture from STEP 7.

2. Transfer 1.5 mL of culture into a new 1.5 mL centrifuge tube. Label this tube “plasmid DNA” with your candidate number.

3. Use this sample to isolate plasmid DNA according to the STEP 1 procedure described in this manual. Store samples as -20°C.

**-------------------------------------------------------------------------------------------------------------------**

**STEP 9: RESTRICTION DIGESTION OF PLASMID DNA. AGAROSE ELECTROPHORESIS; DNA SEQUENCING REACTION**

**Purpose:** To determine size of the GFP-positive plasmid

Restriction Digestion Set-Up

1. Label a 1.5 mL microfuge tube with your candidate number and “pPEM109-GFP +Xba1” and proceed to set up restriction digestion as described in the table below:

*HINT: Put a checkmark* ✓ *next to protocol reagents “Reagent added?***”** *as you add them to help you remember what has been added to each tube.*

| **Reagents** | **Restriction enzyme**  **reaction**  **(uL)** | **Reagent added? (**✓**)** |
| --- | --- | --- |
| ddH_2_0 | 8.4 |  |
| 10X Buffer | 1.6 |  |
| pPEM109 plasmid DNA (from **STEP 8**) | 5.0 |  |
| XbaI enzyme | 1.0 |  |
| **Total:** | **16** |  |

2. Label a 1.5 mL microfuge tube with your candidate number and “pPEM109 +Xba1” and set up:

| **Reagents** | **Restriction enzyme**  **reaction**  **(uL)** | **Reagent added? (**✓**)** |
| --- | --- | --- |
| ddH_2_0 | 8.4 |  |
| 10X Buffer | 1.6 |  |
| pPEM109 plasmid DNA (from **STEP 1**) | 5.0 |  |
| XbaI enzyme | 1.0 |  |
| **Total:** | **16** |  |

3. Incubate digestions at 37°C for 1 h. Add 4 uL of DNA sample loading dye to stop reaction.

Why is it important to compare STEP 1 results with STEP 8? What do you expect and why?

DNA Sequence Analysis:

Remove 12 ul of STEP 8 plasmid DNA and place it in a PCR striptube tube containing 3 ul of GFP DNA sequencing primer (5 uM) as directed by your instructor. DNA sequencing samples will be sent to an outside sequencing lab for sequence determination. We will analyze these results when the sequence becomes available.

DNA gel electrophoresis:

1. Load 8 ul of XbaI-digested DNA from both the STEP 8 and the STEP 1 DNA samples. Each student should have 2 samples to load onto the gel. Depending on gel lane availability, your instructor may also have you load an undigested control DNA sample. Run the gel as described in STEP 3. When the gel finishes, image the gels using BIO-RAD GelDoc.
