# Supplementary material for: A simple, robust, broadly applicable insertion mutagenesis method to create random fluorescent protein: target protein fusions
Source: G3 (Bethesda). 2024 Feb 16;14(5):jkae036. doi: 10.1093/g3journal/jkae036 (PMC11075570; doi:10.1093/g3journal/jkae036)
Supplement: jkae036_Supplementary_Data [file jkae036_supplementary_data.zip › Supplemental_Tables_G3-2024-404874.docx]

**Table S1.** Summary of all insertion points of GFP ORFs into the *fusA* ORF that encodes EF-G. The insertion site structure column provides a description of the structural elements found at the insertion site. The crystal structure of *E. coli* ribosome-bound EF-G was employed for insertion site structure analysis using PDB ID code 4KIY. These results are from a collection 70 candidate plasmids analyzed by DNA sequencing from two different transposition experiments. The transformations of each transposition were standardized to produce 2,000 transformants per plate. Ten plates were produced in each transformation.

| EF-G codon | Insertion Domain | Insertion Site Structure |
| --- | --- | --- |
| 35 | GTP-binding domain switch I region | Residues 35 - 65 loop at protein surface, residues 38-46 unresolved |
| 43 | GTP-binding domain switch I region | Residues 35 - 65 loop at protein surface, residues 38-46 unresolved |
| 49 | GTP-binding domain switch I region | Residues 35 - 65 loop at protein surface, residues 38-46 unresolved |
| 56* | GTP-binding domain switch I region | Residues 35 - 65 loop at protein surface, residues 38-46 unresolved |
| 312 * | Connector between GTP-binding domain and Domain II | Long loop sequence 285-316 |
| 380* | Surface exposed, Domain II | 380 single amino acid break between two short beta-strands |
| 410* | Connector between Domain II and Domain III | Long surface loop sequence 400 to 415 |
| 415* | Connector between Domain II and Domain III | Long surface loop sequence 400 to 415** |
| 464 out of  Frame*,** | Domain III | At start of a helix |
| 484 | Connector between Domain III and Domain IV | Surface connector sequence between domains |
| 503 | Domain IV | Surface exposed in short beta-strand |
| 528* | Domain IV | 9 amino acid surface exposed loop 526 to 534 |
| 533 | Domain IV | 9 amino acid surface exposed loop 526 to 534 |
| 590 | Domain IV | Residues 583 to 589 surface exposed loop at tip of Domain IV, 590-592 at the start of exposed alpha helix |
| 644 | Domain V | 8 amino acid surface exposed loop 644 to 651 |
| 695 | Domain V | Surface exposed terminal residues of 6 residue alpha helix (692 to 697)*** |

*These EF-G insertion sites were obtained only once; all other insertion sites were obtained multiple times.

**The fused ORF “464 out of frame” should contain codons 464 - 466 flanking both insertion junctions. The upstream sequence is correct, but the downstream insertion site is missing the first nucleotide of codon 464. Therefore, the chimeric ORF produces a protein that contains codons 1 to 463 from EF-G with a C-terminal GFP fusion.

***There are 704 amino acids in EF-G, therefore this fused ORF is equivalent to a C-terminal GFP-fusion.

**Table S2.** Summary of all insertion points of GFP ORFs into the target ORFs Era and EngA. The insertion site structure column provides a description of the structural elements found at the insertion site. The crystal structure of *E. coli* Era (PDB ID 3IEU) was employed for insertion site structure analysis, but the structure of *E. coli* EngA has not been determined. These data are from one transposition for each target ORF. The transformations of each transposition were standardized to produce 2,000 transformants per plate. Ten plates were produced in each transformation. Ten insertions were identified in the EngA ORF and three in the Era ORF.

| Target ORF  (Number of independent isolates) | Insertion Site | Insertion Domain | Insertion Site Structure |
| --- | --- | --- | --- |
| Era (1) | 129 | G-IV sequence motif of GTP-binding domain. | 7 residue surface loop |
| Era (1) | 177 | Inter-domain linker. | 13 residue surface loop |
| Era (1) | 178 | Inter-domain linker. | 13 residue surface loop |
|  |  |  |  |
| EngA (1) | 181 | Immediately after end of GTP-binding domain I. | ND |
| EngA (5) | 189 | Inter-domain acidic linker. | ND |
| EngA (1) | 197 | Inter-domain acidic linker. | ND |
| EngA (2) | 198 | Inter-domain acidic linker. | ND |
| EngA (1) | 262 | GTP-binding domain 2. | Surface loop* |

*This insertion is near a highly conserved surface loop in involved in nucleotide binding in all GTPases (the G-III motif DTXG).

**Table S3.** Summary of all insertion points of GFP ORFs into the target ORFs FtsZ and MreB. An exhaustive structural analysis of the insertion sites was not undertaken. To increase the screening capacity two transposition reactions were pooled, and transformants were plated on large petri dishes (150 mm x 15 mm). The transformations were standardized to produce 20,000 transformants per plate. Six plates were produced in each experiment representing a screen of a total of 120,000 colonies per ORF. These data are compiled from using this approach on two separate occasions. Overall, ten insertions into FtsZ and fourteen into MreB were documented.

| Target ORF  (Number of independent isolates) | Insertion Site |
| --- | --- |
| FtsZ (2) | 5 |
| FtsZ (1) | 25 |
| FtsZ (1) | 27 |
| FtsZ (2) | 36 |
| FtsZ (1) | 41 |
| FtsZ (1) | 57 |
| FtsZ (1) | 66 |
| FtsZ (1) | 170 |
| FtsZ (1) | 334 |
| FtsZ (1) | 340 |
|  |  |
| MreB (1) | 15 |
| MreB (1) | 37 |
| MreB (1) | 41 |
| MreB (1) | 59 |
| MreB (3) | 61 |
| MreB (1) | 75 |
| MreB (1) | 97 |
| MreB (1) | 151 |
| MreB (1) | 228 |
| MreB (3) | 235 |
